# Comparing clinical outcomes in patients with diabetes undergoing coronary artery bypass graft and percutaneous coronary intervention in real world practice in Iranian population

**DOI:** 10.1186/s12872-022-02521-z

**Published:** 2022-03-03

**Authors:** Arezo Arabi, Behshad Naghshtabrizi, Hamid Reza Baradaran, Yousef Moradi, Mohsen Asadi-Lari, Ali Mehrakizadeh

**Affiliations:** 1 Department of Cardiology, School of Medicine, Hamedan University of Medical Sciences, Hamedan, Iran; 2 Endocrine Research Center, Institute of Endocrinology and Metabolism, Iran University of Medical Sciences, Tehran, Iran; 3 Ageing Clinical and Experimental Research Team, Institute of Applied Health Sciences, University of Aberdeen, Aberdeen, UK; 4 Department of Epidemiology and Biostatistics, Faculty of Medicine, Kurdistan University of Medical Sciences, Kurdistan, Iran; 5 Department of Epidemiology, School of Public Health, Iran University of Medical Sciences, Tehran, Iran; 6 Cardiology Department, Imam Khomeini Hospital Complex, Tehran University of Medical Sciences, Tehran, Iran

**Keywords:** Clinical outcomes, Diabetes, Coronary artery bypass graft, Percutaneous coronary intervention

## Abstract

**Background:**

Coronary artery bypass graft (CABG) is generally regarded as one the treatment options for coronary artery disease (CAD) in patients with diabetes. In recent years, with the advent of drug-eluting stents (DES), percutaneous coronary intervention (PCI) was introduced as a suitable alternative for CABG. The aim of this study was to compare the incidence of major adverse cardiac and cerebrovascular event (MACCE) during mid-term period in patients with diabetes treated with 2 revascularization strategies.

**Methods:**

This historical cohort study was conducted on 750 consecutives patients with diabetes in a single cardiovascular center from July 2009 to March 2012 in Iran. We included previously known case of DM treated with antidiabetic medications (with or without end organ damage) and patient with preoperational evaluation FBS test > 126 (not on the day of the surgery) who were revascularized by 2 strategies. We excluded those patients whose follow-up was not possible.

**Results:**

Finally, out of 697 eligible patients, 355 patients underwent a CABG and 342 underwent a PCI: 53 patients were lost to follow-up (27 in CABG and 26 in PCI groups). The mean follow-up time was 900.68 ± 462.03 days in the CABG and 782.60 ± 399.05 in PCI groups. There were 17 (9.13%) cardiac deaths in the CABG group and 8 (4.45%) in the PCI group; this difference was not significant (*P* = .11). There was 14 (7.58%) cerebrovascular accident in the CABG group and 4 (2.31%) in the PCI group; this difference was significant (*P* = .04). Moreover, the frequency of the target vessel revascularization in the CABG and PCI groups was 6 (3.32%) and 31 (17.11%) (*P* < .001), respectively. Myocardial infarction in the CABG group was 5 (2.77%) and 14 (7.86%) in the PCI group (*P* = .009). Finally, the frequency of MACCE in the CABG and PCI groups was 41(20.70%) and 47(24.16%) respectively; this difference was not statistically significant (*P* = .195).

**Conclusion:**

Patients with CABG in this study experienced more CVA, while the frequency of TVR and non-fatal MI was higher in the PCI arm.

## Introduction

Individuals with diabetes experience a higher rate of mortality and morbidity from coronary artery disease (CAD) than with patients without diabetes [1, 2]. Increasing number of patients with diabetes will reach to 360 million by 2030 while 3/4 of them will be in developing and non-developed countries [3, 4]. Diabetes worsen outcomes of patients following both medical and invasive treatment strategy compared with non-patients with diabetes [5, 6]. Association of metabolic disorders with DM led to accelerated atherosclerotic progression and complexity of coronary lesion [7, 8]. Concurrent with the epidemiological transition, the increasing amount of diabetes as a major risk factor for CAD and decision-making for treatment strategy have raised much concern in clinicians in recent decades. Revascularization with 2 methods of coronary artery bypass graft (CABG) and percutaneous coronary intervention (PCI), as treatment options for this high risk subgroup of patients, has attracted much attention recently, as one-fourth of 1.5 million revascularizations being performed annually involve patients with diabetes [9, 10]. Hence, in recent years, many studies have been conducted to compare the clinical outcomes of these 2 methods. A marked reduction in the difference between clinical outcomes of the 2 methods is seen following introduction of drug-eluting stents (DES) and new oral antiplatelet drugs [11–13]. Concurrent with introducing DES and reduction in-stent restenosis and repeat revascularization, compared with bare-metal stenting, the best revascularization strategy for patients with diabetes with multivessel CAD remains to be under despot [14]. We achieved this historical cohort study to compare the clinical outcome of CABG and PCI in patients with diabetes with multivessel CAD. The results of this retrospective study could be effective to determine the most appropriate revascularization strategy for Iranian patients with DM.

## Methods

This study was a historical cohort study in a single cardiovascular center to evaluate the incidence of major adverse cardiac and cerebrovascular events (MACCE) in patients with diabetes and multi-vessel coronary disease.

### Patient selection

We recruited all the patients from a main cardiovascular data base of Ekbatan hospital, Hamadan, Iran. The study was designed in accordance with the principles of the declaration of Helsinki and got approval from the local ethics committee of our hospital (Ekbatan Hospital). We included in this study every patient with diabetes and significant coronary artery disease (more than 70% stenosis in one major coronary artery), undergoing elective revascularization by CABG or PCI from July 2009 (when for the first time PCI was available in our center by interventional cardiologists) to March 2012. We excluded patients with Left main disease, ESRD patients, poor prognosis patients, such as those with malignancy, cardiogenic shock, ACS setting patient during 24 h before revascularization (including STEMI, non-STEMI Unstable angina), concomitant valve surgery, and previous CABG or PCI; and those with anatomical problems, such as atrial septal defect, ventricular septal defect, and mitral valve sever regurgitation. Thus, we had 572 patients with diabetes (previously known case of DM treated with antidiabetic medications (with or without end organ damage) and patient with preoperational evaluation FBS test > 126 (not on the day of the surgery). All patients signed informed consent to undergo CABG and PCI. Because the study was retrospective, we were unable to obtain informed consent from those patients in whom MACCE occurred, we also obtained an informed consent waiver from the same ethics committee.

### PCI and CABG

Decision-making about the revascularization strategy was done after consult with surgical services, with attention to the patient's preference. Patients with complex diseases, such as LAD involvement, multivessel disease, severe left ventricular dysfunction, and diabetes, were referred for CABG. Also, PCI with DES stents (drug eluting stent) in patients with diabetes was preferred over bare metal stents (BMS). PCI was often achieved with femoral approach of the Seldinger technique. All patients in the PCI group received 600 mg clopidogrel and 325 mg aspirin during 24 h before the intervention. In-group cardiac enzymes and electrocardiograms were checked during the first 24 h after the intervention routinely.

In the CABG group, on pump CABG was preferred to off pump CABG because of graft patency and left internal mammary artery (LIMA). All patients were monitored for at least 72 h after surgery at the intensive care unit and antiplatelet agents, such as aspirin and clopidogrel, were not administered 48 h before surgery. After surgery, heparin was prescribed routinely for all patients.

Patients in the PCI group received dual antiplatelet therapy for at least 6 months if BMS was implanted and 1 year if DES was implanted, after which aspirin monotherapy continued. The group of CABG patients received standard low-dose aspirin started within 6 h after surgery plus clopidogrel for 2 months and then continued aspirin indefinitely[15, 16].

During the first year after the index procedure, all patients were visited at intervals of one to three months, either at the onset of new cardiovascular signs and symptoms (chest pain, shortness of breath, stroke-suggesting symptoms) or hospitalized in the emergency department. The diagnosis of myocardial infarction was made and recorded according to the third universal definition of MI.; and for the subsequent years, this visiting interval was adjusted by clinicians taking into account the patient’s condition.

### Clinical outcomes and follow-up

The outcome in this study was MACCE, including non-fetal myocardial infarction (MI), cardiac death, cerebrovascular accident (CVA), and target vessel revascularization (TVR). We assessed the occurrence of MACCE based on the information provided by telephone contact, hospital readmission, and clinical records. If the follow-up was impossible, it was considered as loss to follow-up. Diagnose of cardiac death was based on the main cause of death registered on the death certification and other clinical events determined by the attendance. Cerebrovascular events were defined as strokes and transient ischemic attacks. Post procedural medical treatment was assessed via telephone interviews. By the end of the study time those who did not experience any outcome were considered as censored.

### Statistical analysis

The values are presented in mean ± standard deviation, which were compared using an independent *t* test and frequency, which are tested using the χ^^2^ test for continuous and categorical variables, respectively. The cumulative clinical event rate during follow-Up at 1 and 3 years in CABG and PCI groups were compared using the log-rank test. After that, a Cox proportional hazards regression model was applied to find the significant predictors of MACCE. In this model hypertension, stroke, and MI history were adjusted. After determining the best model according to the presence or absence of the predictors based on the Akaike Information Criterion using the stepwise method, the proportionality of hazards was checked using Schoenfeld residuals.

## Results

**Fig. 1:**
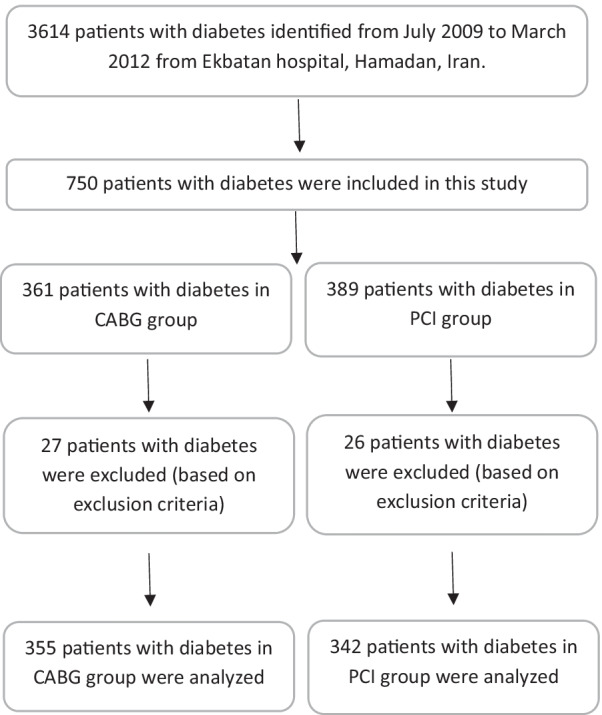
Flow diagram of the study population

After evaluating 3614 clinical records of those who underwent revascularization in this center, 750 patients with diabetes who met our study criteria were included in this study. We had 53 (9.28%) losses to follow-up (27 in CABG arm and 26 in PCI arm). Finally, the study was conducted with 676 patients with diabetes (334 in CABG arm and 342 in PCI arm) (Fig. 1). The mean follow-up time was 891.45 ± 458.34 days in the CABG and 790.96 ± 415.21 in the PCI groups. Baseline clinical characteristics of the patients are shown in Table 1. In brief, the frequency of ejection fraction (EF) < 0.40 was not significantly different between PCI and CABG groups. Also, the mean EF was significantly higher in the PCI group than in the CABG group. The proportion of peripheral vascular disease, LAD involvement, number of treated vessels, left ventricular (LV) dysfunction, insulin dependence, consumption of clopidogrel, number of diseased vessels, and the amount of stent/graft per patient were significantly higher in the CABG group compared with the PCI group (Table 1).

**Table 1:** Baseline clinical characteristics of patients

Variable	CABG	PCI	*P* value
	(n = 355)	(n = 342)	
Age (mean ± SD)	61.68 ± 8.9	61.42 ± 10.6	0.720
EF	0.474 ± 0.07	0.490 ± 0.09	0.002
EF (< 0.40), n(%)	38 (11.38)	22 (12.43)	0.722
Male, n (%)	186 (55.7)	98 (52.9)	0.552
Hypertension, n (%)	200 (59.8)	116 (62.7)	0.527
Hyperlipidemia, n (%)	143 (42.81)	85 (45.95)	0.491
Smoking, n (%)	71 (21.26)	24 (24.32)	0.424
Peripheral vascular disease (%)	49 (14.67)	10 (5.41)	0.001
MI history, n (%)	82 (24.55)	42 (22.70)	0.635
Stroke history, n (%)	9 (2.69)	3 (1.62)	0.423
LAD involvement, n (%)	334 (100)	161 (87.3)	< 0.001
Treated vessel territory, n (%)			
RCA	258 (77.25)	67 (36.22)	< 0.001
LAD	319 (95.51)	116 (62.70)	< 0.001
LCX	238 (71.26)	49 (26.49)	< 0.001
LV function, n (%)			
Mild	160 (42.90)	51 (27.57)	< 0.001
Moderate	27 (8.08)	12 (6.49)	
Severe	9 (2.69)	9 (4.86)	
Normal	138 (41.32)	113 (61.08)	
Treated/untreated diabetes	308 (92.22)	160 (86.49)	0.039
Treatment of diabetes			
Insulin	52 (14.6)	39 (11.4)	0.397
Oral drug	191 (53.8)	180 (52.6)	
Dietary	83 (23.4)	86 (25.1)	
Nothing	29 (8.2)	37 (10.8)	
Aspirin (%)	349(98.3)	340 (99.4)	0.171
Clopidogrel (%)	301(84.8)	328 (95.9)	< 0.001
No. of disease vessels, n (%)			
One vessel disease	21 (5.9)	157 (45.9)	< 0.001
Two vessel disease	77 (21.7)	141 (41.2)	
Three vessel disease	257 (72.39)	44 (12.87)	
Number of stent/graft per patient, n (%)			
1	33 (9.30)	261 (76.32	
2	105 (29.58)	73 (21.35)	
3	171 (48.17)	8 (2.34)	
4	44 (12.39)	0 (0%)	
5	2 (.56)	0 (0%)	

The procedural characteristics of the patients are presented in Table 2. Table 2 shows that 94.6% of patients with CABG had the on-pump type, 78.1 of patients with CABG had LIMA type of graft on LAD, 84.7 had DES type of stent, and 64.9 had EES type of drug eluting stent.

**Table 2:** Procedural characteristics of patients

Variable	N (%)
Type of CABG, n (%)	
On Pump	316 (94.6)
Off Pump	18 (5.4)
Type of graft on LAD, n (%)	
LIMA on LAD	261 (78.1)
SVG on LAD	73 (21.9)
Type of drug eluting stent, n (%)	
EES	96 (64.9)
PES	25 (16.9)
ZES	6 (4.1)
BES	21 (14.2)
Type of stent DES/BMS*	
DES stent	145 (84.7)
BMS stent	26 (15.3)

Indicates that patients who implemented both DES & BMs were excluded

The cumulative clinical event rate during the follow-up at 1 and 3 years and the comparison between the 2 groups are depicted in Table 3. During the follow-up time, the rate of cardiac death and CVA were not significantly different in the CABG and PCI groups. The rate of TVR was significantly lower in the CABG group in 1 and 3 years of follow-up. Also, the rate of non-fetal MI was higher in PCI significantly in the 3-year follow-up. The overall rate of MACCE was not different significantly in the CABG and PCI groups (Table 3).

**Table 3:** Cumulative clinical event rate during follow-up at 1 year and 3 years

Event	CABG % (n)	PCI %(n)	*P* Value
Cardiac death			
0–1 year	2.79 (9)	2.24 (4)	0.473
0–3 years	5.61 (13)	3.43 (4)	0.184
CVA			
0–1 year	1.47 (5)	0 (0)	0.068
0–3 years	4.85 (13)	2.58 (3)	0.137
TVR			
0–1 year	1.56 (5)	6.06 (11)	0.027
0–3 years	2.63 (6)	16.80 (21)	< 0.001
MACCE			
0–1 year	6.40 (21)	8.15 (15)	0.673
0–3 years	14.04 (34)	21.78 (28)	0.269
MACCE ex TVR			
0–1 year	4.91 (16)	4.43 (8)	0.623
0–3 years	10.95 (28)	12.29 (15)	0.667
Non-fetal MI			
0–1 year	0.62 (2)	2.80 (5)	0.095
0–3 years	1.32 (3)	7.56 (9)	0.006

The clinical outcome of the follow-up after stenting during the study period and comparing DES versus BMS and DES versus CABG are shown in Table 4. The results showed that the rate of clinical outcome for cardiac death and MACCE without TVR were higher in the CABG group compared with DES, while the rate of TVR was significantly higher in DES compared with CABG. Moreover, the MACCE, cardiac death, CVA, TVR, non-fetal MI, and MACCE without TVR rates were significantly higher in BMS compared with DES (Table 4).

**Table 4:** Clinical outcome of follow-up after stenting during study period

	DES (n = 145), n (%)	BMS (n = 26), n (%)	*P* value*	CABG (n = 334), n (%)	*P* value**
Cardiac death	0 (0)	2 (14.28)	< 0.001	15 (8.59)	0.014
CVA	1 (1.36)	2 (14.28)	0.041	14 (7.58)	0.063
TVR	12 (15.28)	7 (42.42)	0.013	6 (3.52)	< 0.001
MACCE	14 (17.61)	9 (51.42)	0.001	39 (20.91)	0.757
MACCE ex TVR	4 (5.36)	6 (37.50)	< 0.001	32 (19.5)	0.020
Non-Fatal MI	3 (4.05)	3 (20.68)	0.029	5 (4.12)	0.439

*The comparison between DES versus BMS

**The comparison between CABG versus DES

**Fig. 2:**
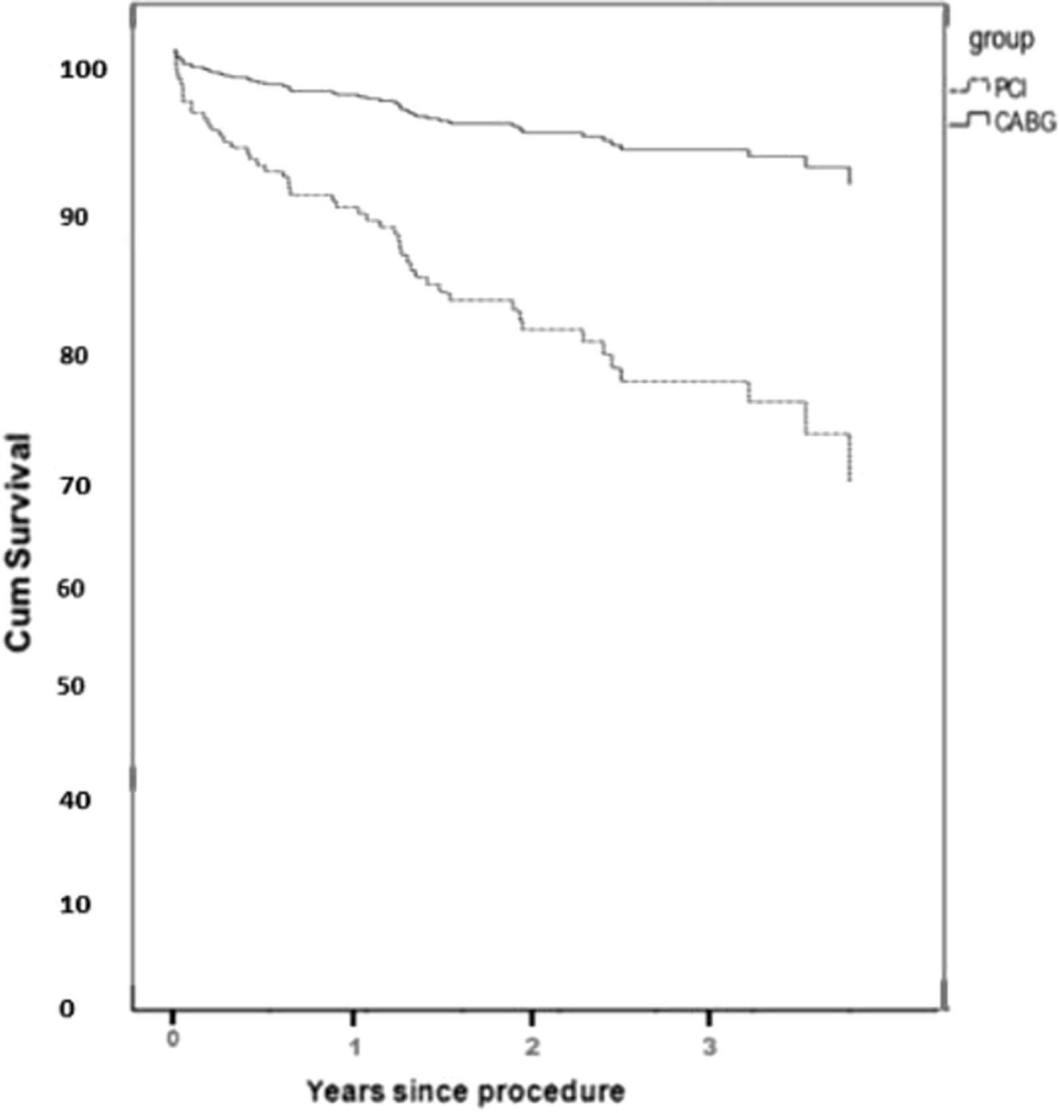
Survival function of MACCE

Table 5 shows the results of the cox proportional hazards regression for MACCE. Using the stepwise method, hyperlipidemia, age (> 60), treatment modality (PCI versus CABG), LAD involvement, left ventricular dysfunction, clinical presentation, and complete revascularization were selected as the best predictors. The Schoenfeld residual test resulted in the proportionality of hazards for the predictors. Propensity score matching was applied on the covariates using the treatment modality as the response for the logistic regression. After forcing the matching into the cox regression model, the results showed that patients with hyperlipidemia were 2.04 (95% CI; 1.15–3.59) more likely to get MACCE compared with patients without hyperlipidemia, this was 3.07 (95% CI; 1.56–9.09) for patients in PCI arm compared with CABG (Fig. 2), and 4.54 (95% CI; 1.65–12.48) for patients with severe left ventricular dysfunction compared with healthy people (Table 5).

**Table 5:** Results of cox regression to predict time to MACCE adjusted based on hypertension, stroke and MI history

Variable	Adjusted Hazard ratio	CI 95% (HR)	Significance
Age (> 60)	1.67	0.90–3.09	NS
Dyslipidemia (yes)	2.04	1.15–3.59	S
Group (PCI)	3.07	1.56–9.09	S
LAD involvement	4.35	0.58–12.27	NS
Normal LV dysfunction	Ref	–	
Mild LV dysfunction	1.52	0.78–2.94	NS
Moderate LV dysfunction	1.89	0.73–4.89	NS
Severe LV dysfunction	4.54	1.65–12.48	S
Type setting	1.66	0.90–3.12	NS
Complete (yes)	2.18	0.96–4.91	NS

NS: Not significant, S: Significant

## Discussion

This study showed that there was no statistically significant difference in the unadjusted incidence of MACCE between PCI and CABG groups. In contrast, the adjusted analysis using the cox PH model depicted a hazard ratio of 3.07 for the PCI group relative to CABG for the incidence of MACCE. According to this study, the incidence rate of CVA was similar in 2 arms of CABG and PCI. In most of the previous studies, the lower rate of MACCE in CABG has been demonstrated versus PCI groups [17–20].

In a 5-years follow-up trial by Contini et al. [21] in 2012, the incidence of MACCE was higher in the PCI group than the CABG group. Also, Tartaniti et al. [22] showed that in patients with diabetes and multi-vessel coronary disease, survival rate was similar in the PCI and CABG groups. In an evaluation of a difference between CABG and PCI in patients with diabetes and multi-vessel coronary disease, Hee et al. [23] depicted that the rate of MACCE occurrence in PCI is statistically higher than in CABG (29% vs. 14%; *P* = 0.016). Deamen et al. [24] in their study “Arterial Revascularization Therapies (ARTS), part I and II” demonstrated a higher occurrence of MACCE in PCI (47.3%) than in CABG (17.7%), while the results for CVA was vice versa (5.4% vs. 6.3%). In a systematic review of comparing CABG vs PCI programmed by Saswata et al. [25], patients with diabetes generally had better outcomes with CABG than with PCI (18.7% vs. 26.6%; *P* = 0.005) as well as cardiac events. In a meta-analysis evaluating the incidence rate of MACCE in patients with diabetes and multi-vessel coronary disease by Fan zhang et al. [26], a 12% reduction was detected in the CABG group. After adjusting for the effect of group, being as CABG or PCI in the Cox PH model, we found that the hazard of MACCE occurrence in patients with PCI is 3.07 more likely than CABG arm, which is significant. The results of the Cox PH model showed that patients with dyslipidemia were 2.04 more likely to experience MACCE compared with patients without dyslipidemia. The results also showed that patients with severe LV dysfunction experienced MACCE 4.54 times more than those with normal LV function.

Our study demonstrated that the incidence rates of cardiac death during 1 year and 3 years follow- up were not statistically different between CABG and PCI groups. In a study to find the optimal coronary revascularization policy in patients with diabetes, Kamalesh et al. [27] showed that there was no difference in the survival rate of cardiac death between the 2 arms. To compare medical therapy, PCI, and CABG as 3 therapeutic strategies for stable coronary disease in 2013, Lima et al. [28] found cardiac mortality rates of 18.8% and 12.5% for PCI and CABG, respectively. In an editorial, review, which was a randomized clinical trial comparing CABG versus PCI, conducted by Emmanuel Moss et al. [29], MACCE was significantly higher with PCI compared with CABG, as well as cardiac death in patients with diabetes and multi-vessel coronary disease in 2013.

In our study, the incidence rates of TVR were statistically different in the 2 arms at 1- and 3-year follow-up and was lower in the CABG group. Bhatt ad topol in a study “The Arterial Revascularization Therapy Study (ARTS) and the Stent or Surgery (SoS)” demonstrated that the rate of TVR was significantly lower in CABG [30]. A similar result was found by Contini et al. [21]. The incidence rate of non-fetal MI was significantly different between the 2 groups in the 3-year follow-up, while the survival rate was similar in these 2 groups during the 1-year follow-up. A similar result was found in another study [27]. Non-fetal myocardial infarction incidence rates were not significantly different in patients with PCI or CABG in the study of Leia et al. [23].

Comparing the incidence rate of MACCE in DES and BMS, cardiac death, CVA, TVR, MACCE ex TVR, and non-fetal MI, our results demonstrated an overall significant reduction in the occurrence rate of MACCE in DES versus BMS groups. In the ERACI III registry planned by Ong et al. in 2007 in Argentina, the 3-year MACCE was significantly lower in DES compared with BMS [31] and similar results are found in the study of Contini et al. [21]. The results from Kapur et al. in 2009 showed no significant differences between DES and BMS in CVA and non-fetal myocardial infarction incidence rates [19]. In a meta-analysis of 14 trials comparing CABG and DES in patients with diabetes and multi-vessel coronary artery disease, De Luca et al. [32] in 2014 showed that CABG reduces the incidence rate of TVR compared with DES, while a lower rate of MACCE was found for CABG than DES. A similar result was driven by Wander and Chhabra in 2010, as the clinical benefits of DES versus BMS at 12 months showed that the incidence rate of TVR was significantly reduced; also, in a study by Xiaolong et al. [33] it was found that CABG can significantly reduce the rates of myocardial infarction than DES in patients with diabetes and multi-vessel coronary disease.

The present study showed no difference in the occurrence of MACCE compared with CABG and DES and CVA and non-feta MI, while a statistical difference was depicted in the occurrence of cardiac death comparing CABG and DES (0% vs. 8.59%; *P* = 0.014) as well as for TVR (15.28% vs. 3.52%; *P* < 0.001).

Comparing the differences between DES and bilateral internal thoracic artery grafts in patients with diabetes in 2012, Moshkovitz et al. [34] demonstrated that MACCE survival rate in patients with diabetes who were revascularized by CABG was better than PCI with DES. In 2 other studies performed by Javaid et al. in 2007 and Qiao et al. in 2009, it was found that clinical outcome and MACCE rate in patients with diabetes multi-vessel coronary disease were higher in PCI with DES than the CABG strategy [35, 36]. The occurrence of cardiac death was higher in CABG compared with DES. While there was no significant difference between the 2 arms of CABG and DES in our study, a higher rate of CVA incidence was found in CABG compared with DES in Qiao et al. [36]. In our study, the survival rate of TVR in CABG and DES was 3.52 and 42.42, respectively. Similar results were found in Moshkovitzh et al. [34] study where the hazard ratio of TVR was 7 in DES likely than CABG. Qiao et al. [36] showed that the incidence rates of non-fetal MI in patients with CABG and DES were statistically the same during the study period.

Although present study cannot show superiority of one revascularization plan, but it shows results and follow-up of real practice based on expert consensus on decision making for revascularization of diabetic patients.

At the time period of this study, HbA1c and BNP levels of patients were unavailable, also SYNTAX score calculation was not performed at the time of treatment planning, due to unavailable laboratory facilities and strong guideline recommendation for SYNTAX score calculation at study conduction time. So our study has important limitations, and we suggest studies considering SYNATX score and HbA1c levels.

45% of patients in PCI arm had single vessel disease comparing with 6% in CABG arm, and it was a major limitation of our study.

## Conclusion

Patients with CABG in this study experienced more CVA, while the frequency of TVR and non-fatal MI was higher in the PCI arm.

## Data Availability

Data are available and can be accessed from the corresponding author with reasonable inquiry.

## Abbreviations

CI: Confidence interval
CAD: Coronary artery disease
CAD: Coronary artery disease
CABG: Coronary artery bypass graft
PCI: Percutaneous coronary intervention
DES: Drug-eluting stents
MACCE: Major adverse cardiac and cerebrovascular events
ICU: Intensive care unit
CVA: Cerebrovascular accident
TVR: Target vessel revascularization
